# Electrochemically Controlled Deposition of Low‐Crystalline Covalent Organic Frameworks on Nanocarbon Electrode Toward Metal‐Free Oxygen Reduction Electrocatalyst

**DOI:** 10.1002/smll.202410475

**Published:** 2024-12-17

**Authors:** Kosuke Sato, Shinsuke Inagi

**Affiliations:** ^1^ Department of Chemical Science and Engineering Institute of Science Tokyo 4259 Nagatsuta‐cho, Midori‐ku Yokohama Kanagawa 226–8501 Japan

**Keywords:** composite materials, covalent organic frameworks, electrocatalyst, electrogenerated acid, nanostructure

## Abstract

Morphology‐controlled synthesis of covalent organic frameworks (COFs) offers significant potential for electrochemical applications. However, controlling the deposition of nanometer‐scale COFs on carbon supports remains challenging due to the need for a slow COF generation rate and the dispersion of carbon supports in liquid‐phase synthesis. In this study, nanometer‐scale COF/carbon composites are fabricated using electrochemically generated acid (EGA) to assist in the formation of imine‐type COFs, which are then deposited onto pre‐cast nanocarbon supports on an electrode. A monomer combination of tri(4‐aminophenyl)‐1,3,5‐triazine and 2,5‐dimethoxybenzene‐1,4‐dicarboxaldehyde is utilized due to their suitable oxidation potentials, with 1,2‐diphenylhydrazine serving as the EGA source. Through proton generation driven by electrolysis conditions, controlled COF formation is achieved at the single nanometer scale, ranging from 6 to 30 nm, on various nanocarbon supports. The COF/carbon electrode is evaluated as an oxygen reduction reaction (ORR) electrocatalyst, demonstrating superior performance compared to other COF‐based electrode materials containing the 1,3,5‐triazine moiety. The findings experimentally validate the efficacy of the EGA‐assisted COF deposition method for nanostructure construction and its ability to enhance the properties of COF‐based electrodes through morphology tuning.

## Graphical abstract

In this study, a novel synthetic methodology for nanoscale deposition of imine‐type covalent organic frameworks (COFs) using an electrogenerated acid generated at the surface of a nanocarbon electrode is reported. The core‐shell type COF/nanocarbon structure is successfully fabricated by electrolysis condition‐driven deposition, and the composite electrode exhibited a superior performance as a metal‐free oxygen reduction electrocatalyst.

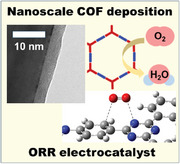

## 1 Introduction

Covalent organic frameworks (COFs) are promising organic materials known for their versatile properties, which stem from their diverse molecular structures and potential to replace traditional functional materials with more sustainable alternatives.^[^ ^1^, ^2^, ^3^ ^]^ The electrochemical applications of COFs have garnered attention due to their durability, diverse electronic structures, and structural tunability. This tunability can endow COF‐based electrodes with valuable properties, such as redox activity for charge storage, adsorption selectivity for sensing, and catalytic activity for facilitating organic reactions.^[^ ^4^, ^5^, ^6^ ^]^ In the realm of metal‐free electrocatalysts, reactions related to electrochemical water splitting, including oxygen reduction reaction (ORR), oxygen evolution, and hydrogen evolution, have been extensively studied.^[^ ^7^, ^8^, ^9^, ^10^, ^11^, ^12^, ^13^, ^14^, ^15^, ^16^ ^]^ However, most studies have predominantly focused on identifying molecular structures that enhance ORR electrocatalytic activity.^[^ ^17^, ^18^, ^19^, ^20^ ^]^ Optimizing electrode morphology, such as increasing surface area and embedding conductive supports at the nanometer scale, is also crucial for improving electrode performance.^[^ ^21^, ^22^, ^23^, ^24^, ^25^ ^]^ By addressing both the molecular design of COFs tailored for ORR and their integration with electrodes at the nanoscale, substantial improvements in ORR catalytic properties can be achieved.

The typical synthesis of COFs with imine bonds is conducted under liquid‐phase conditions, generally involving heating and stirring a reaction mixture that contains monomers and an acid catalyst.^[^ ^26^, ^27^, ^28^ ^]^ In previous studies focused on electrochemical applications, COF‐based composite materials—often comprising COFs and conductive nanocarbons or metal supports—have been fabricated by polymerizing monomers in the presence of these supports.^[^ ^29^, ^30^, ^31^, ^32^ ^]^ Achieving nanometer‐scale COF domains typically requires extremely low monomer concentrations and extended reaction times to avoid the homogeneous nucleation of pure COF phases. Additionally, maintaining uniform dispersion of nanocarbon materials in standard solutions is challenging. These issues can lead to low reproducibility and necessitate lengthy optimization processes to achieve the desired COF/nanocarbon composite structure. Developing a more sophisticated methodology for forming COFs on nanocarbons could significantly enhance electrocatalytic properties.

**Figure 1:**
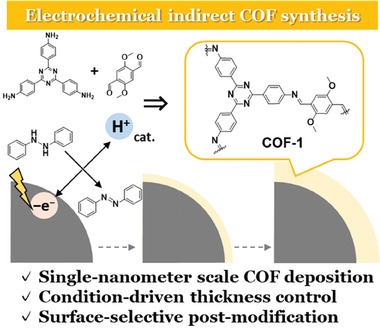
Schematic representation of the proposed electrochemical indirect COF synthesis for controlled deposition.

In our previous work, we demonstrated that electrogenerated acid (EGA) at the electrode surface can serve as a Brønsted acid catalyst for condensation reactions forming imine‐type COFs.^[^ ^33^ ^]^ This cascading electrochemical system enables site‐selective COFs generation directly at the working electrode‐electrolyte interface. Here, we report on an electrochemically controlled synthesis of COFs designed to improve electrocatalytic performance (**Figure** **1**). The controllability of this electrochemical system allows precise tuning of the morphology of COF/carbon composites. The resulting COF/carbon composite electrode exhibited superior performance as an organic ORR electrocatalyst.

## 2 Results and Discussion

### 2.1 Requirements for Monomers

**Figure 2:**
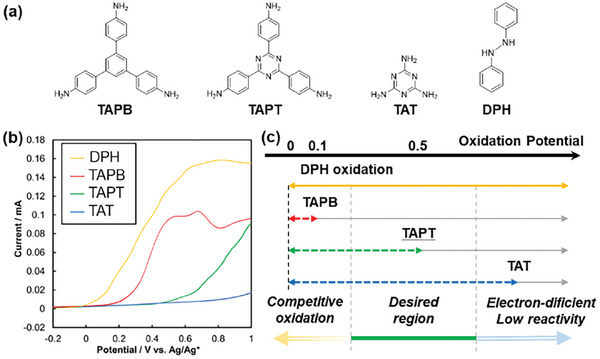
a) Molecular structures of the monomers and the EGA source. b) Linear sweep voltammetry (LSV) of the monomers and the EGA source. c) Schematic summary illustrating the properties of amine monomers and the acceptable oxidation potential used in the electrochemical indirect COF synthesis.

In the proposed EGA‐assisted COF formation method, the choice of monomers was determined by their oxidation potential. 1,2‐Diphenyl hydrazine (DPH) acts as the EGA source to generate protons within the system, with the electrochemical oxidation of DPH on the working electrode being a critical step. The oxidation potentials of typical amine monomers and DPH were investigated using linear sweep voltammetry in a DMF electrolyte (**Figure** **2**a, the detailed experimental procedure was described in the Supporting information). The onset potentials for tri(4‐aminophenyl)benzene (TAPB), tri(4‐aminophenyl)‐1,3,5‐triazine (TAPT), triamino‐1,3,5‐triazine (TAT), and DPH in DMF electrolyte were measured at 0.10, 0.46, 0.79, and −0.02 V versus Ag/Ag^+^, respectively (Figure 2b). To achieve controlled, electrolysis‐driven COF formation, proton generation must be regulated by the current flow, avoiding any competitive oxidation reactions. The range of applicable monomers, based on oxidation potential, is summarized in Figure 2c. The similar oxidation potentials of TAPB and DPH led to competitive oxidation, which hindered controlled proton generation, particularly when the DPH concentration in the electrolyte was lower than that of TAPB. Conversely, electron‐deficient amine monomers such as TAPT and TAT have sufficient oxidation potential gaps, allowing for the selective oxidation of DPH in the electrochemical system. However, when TAT and 2,5‐dimethoxybenzene‐1,4‐dicarboxyaldehyde (DMBDA) were used as monomers, COF formation did not occur at room temperature. The electron‐deficient nature of TAT reduced its reactivity in the imine bond condensation reaction.^[^ ^34^, ^35^ ^]^ The amine monomer with a moderate oxidation potential, TAPT, was therefore anticipated to perform optimally in the proposed EGA‐assisted COF synthesis.

### 2.2 COF Deposition on Nanocarbons

**Figure 3:**
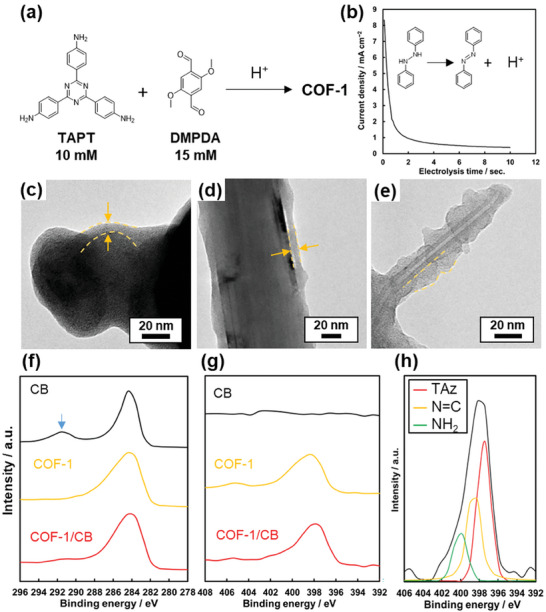
a) Synthesis scheme of TAPT‐DMBDA COF (COF‐1). b) Chronoamperometry profile under electrolysis conditions, with the working electrode set at 0.3 V versus Ag/Ag^+^. c–e) TEM images of COF‐1/CB (c), COF‐1/MWCNT (d), and branched tube in COF‐1/MWCNT. f–h) XPS spectra of CB, COF‐1, and COF‐1/CB composite, focusing on C1s (f), N1s (g), and component analysis for the N1s peak of COF‐1/CB (h). The peak indicated by the blue arrow is attributed to Nafion.

The TAPT‐DMBDA COF (COF‐1) was selected as a model compound for nanoscale morphology control (**Figure** **3**a). The synthesis and identification of COF‐1 using the proposed EGA‐assisted method were preliminarily reported in a previous study.^[^ ^33^ ^]^ Carbon black (CB, Cabot, Vulcan‐XR72) and multi‐walled carbon nanotubes (MWCNT, Showa Denko, VGCF) were used as conductive nanocarbons for the substrate. These nanocarbons were dispersed with Nafion in ethanol and then cast onto a glassy carbon (GC) electrode. Subsequently, the carbon‐modified electrode was immersed in a DMF solution containing 10 mmol dm^−3^ of TAPT, 15 mmol dm^−3^ of DMBDA, 2 mmol dm^−3^ of DPH, and a supporting electrolyte. Electrolysis was typically performed using a constant potential method set at 0.3 V versus Ag/Ag^+^ for 10 s (Figure 3b). The oxidation current displayed a characteristic profile of the constant potential condition, which correlated with proton generation. After washing by immersing the electrode into DMF and drying, the COF‐1/carbon composite film was obtained on the working electrode. Scanning electron microscopy (SEM) images of the COF‐1/CB composite revealed a particle shape, indicating no bulk COF‐1 precipitation (Figure S1, Supporting Information). Transmission electron microscopy (TEM) images showed that the COF‐1/CB composite formed a core‐shell structure (Figure 3c). The COF‐1 shell thickness on CB was 5.9 ± 1.6 nm under the electrolysis conditions. When MWCNT was used as the substrate, the COF‐1/MWCNT composite displayed a coaxial core‐shell structure (Figure 3d), which resulted from the formation of COF‐1 on the MWCNT surface. The COF‐1 shell thickness on MWCNT was 6.2 ± 0.8 nm under the same electrolysis conditions. Additionally, the coaxial core‐shell structure was also observed on branched nanotubes with smaller diameters (Figure 3e). These deposition behaviors suggest that the proposed electrochemical COF deposition method exhibits enhanced adaptability to the substrate structure and can be applied to various nanocarbon materials.

The surface layer of the composite sample was identified as COF‐1 by X‐ray photoelectron spectroscopy (XPS) (Figure 3f–h). The reference COF‐1 sample was synthesized using a conventional method, as described in previous work.^[^ ^36^ ^]^ After electrolysis, the C1s peak of the COF‐1/CB composite became broader, and an N1s peak appeared, compared to the pristine CB film and the reference COF‐1 sample. The N1s spectrum of the COF‐1/CB composite could be deconvoluted into three nitrogen species at binding energies of 397.3, 397.9, and 400.4 eV, corresponding to triazine C─N═C, imine N═C groups, and NH_2_ groups, respectively. The presence of unreacted −NH_2_ groups in the deposited COF‐1 is likely due to the short electrolysis time.

The amount of COF‐1 deposited was estimated using thermogravimetric analysis, with thermal degradation behavior compared to a reference COF‐1 sample (Figure S2, Supporting Information). Based on the separation of weight loss attributed to COF‐1 and carbon black (CB), the COF‐1/CB composite was determined to contain 21 wt% of COF‐1 (Figures S1 and S3, Supporting Information). This value is consistent with the theoretical value of 20%, calculated from the model structure comprising a 60 nm CB core and a 6 nm COF‐1 shell. X‐ray diffraction (XRD) analysis indicated that COF‐1 in the composite had low crystallinity (Figure S4, Supporting Information), which was also corroborated by TEM observations that did not reveal a lattice pattern. The low crystallinity is likely due to the short electrolysis time, as structural rearrangement typically requires several hours.^[^ ^37^, ^38^ ^]^ In addition, the unreacted −NH_2_ group was almost eliminated during the prolonged electrolysis time.^[^ ^33^ ^]^ Due to the low crystallinity, we deduced that the generated COF‐1 did not show significant porosity, although the low generation amount of COF‐1 prevented further investigation.

**Figure 4:**
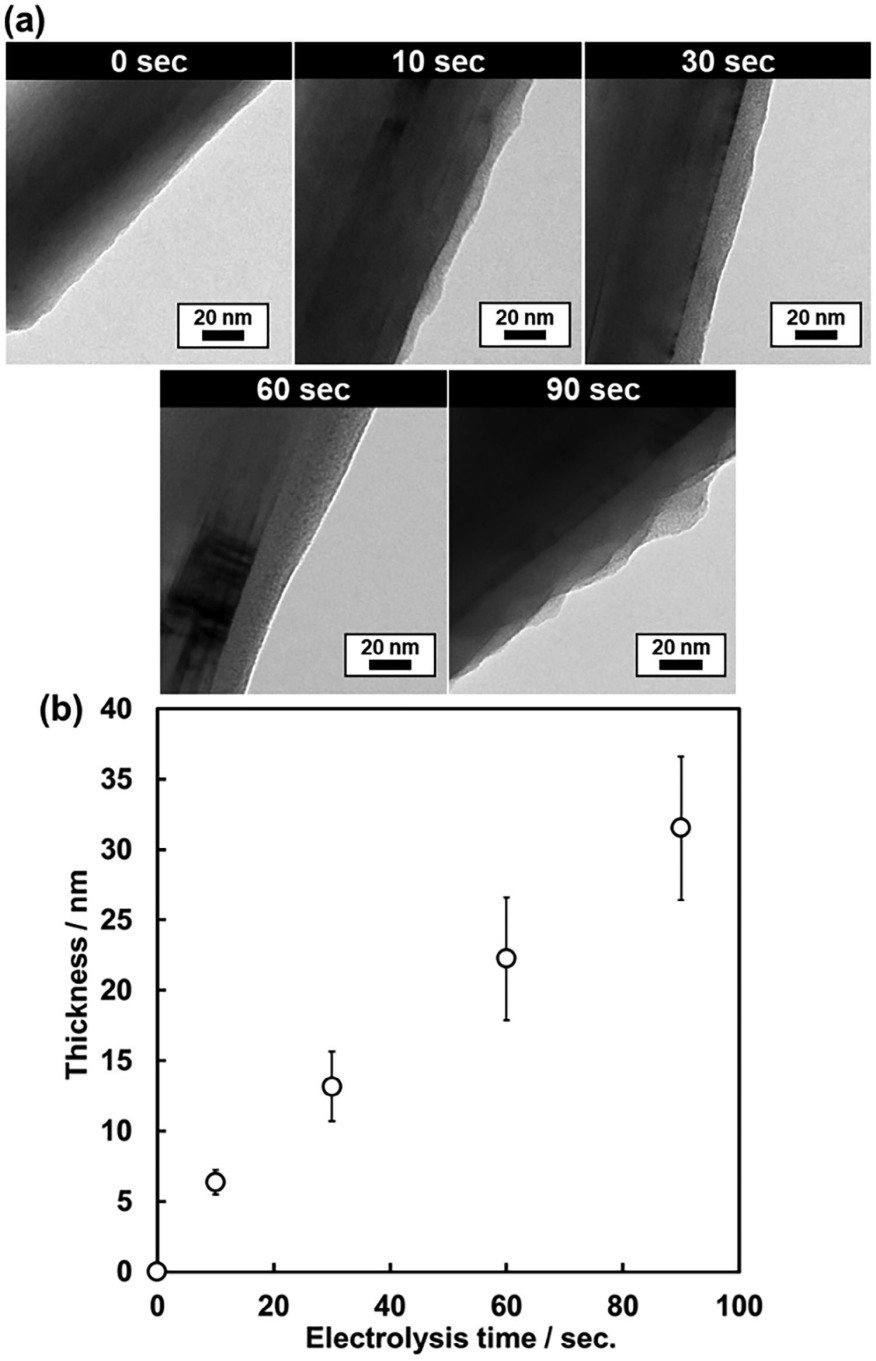
a) TEM images of COF‐1/MWCNT composite fabricated with varying electrolysis times (0–90 sec.). b) Relationship between electrolysis time and COF‐1 layer thickness. The sample size was 20 for each datapoint.

The ability to control COF‐1 thickness was demonstrated by varying the electrolysis time. The coaxial COF‐1/MWCNT composite was selected for this test, as TEM imaging clearly delineates the boundary between the carbon substrate and COF‐1. Electrolysis of COF‐1/MWCNT was conducted at a constant potential of 0.3 V versus Ag/Ag^+^ for 10, 30, 60, and 90 s. The resulting COF‐1/MWCNT composites showed layer thicknesses of 6.2 ± 0.8, 13.1 ± 2.4, 22.2 ± 4.3, and 31.5 ± 5.1 nm, respectively (**Figure** **4** and Figure S5, Supporting Information). The thickness of the COF‐1 layer increased linearly with electrolysis time, indicating a proportional relationship. Since proton generation was more pronounced in the initial phase under constant‐potential conditions, COF‐1 formation was facilitated during the first 10 s. When CB was used as the substrate, the particle diameter of COF‐1/CB also increased systematically with electrolysis time (Figure S6, Supporting Information). These results demonstrate that the proposed EGA‐assisted COF synthesis can control COF thickness from the single‐nanometer scale to 30 nm. Thus, nanometer‐scale control of COF deposition was achieved using this method. Condition‐driven proton generation via an electrochemical procedure is an effective protocol for precise low‐crystalline COF layer deposition Figure 4.

### 2.3 Electrocatalytic Performances

**Figure 5:**
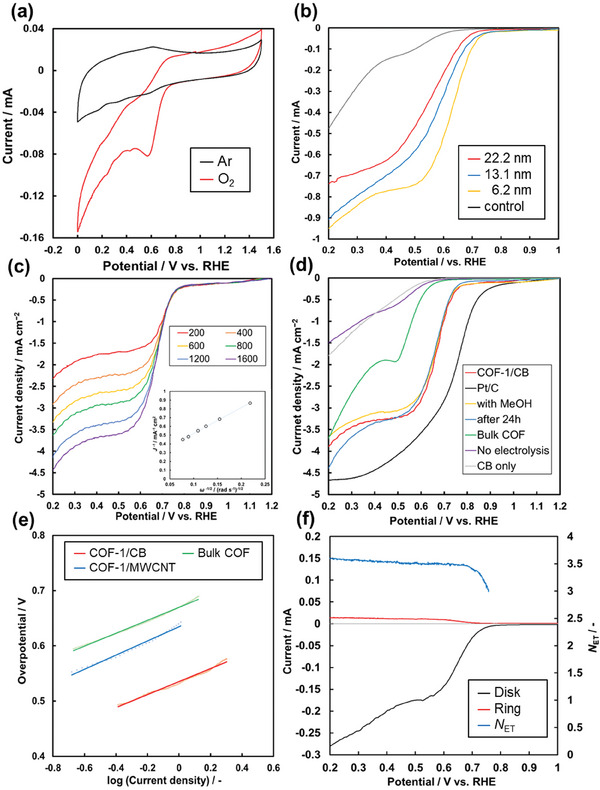
Electrocatalytic ORR properties of the COF‐1 samples. a) CV of the COF‐1/MWCNT electrode in argon and O_2_‐saturated electrolyte. b) LSV of the COF‐1/MWCNT electrode with varying COF‐1 thickness. Control electrode consisting of MWCNT and Nafion (gray line). c) LSV of the COF‐1/CB electrode under different rotating speeds. (inset) Levich plot. d) LSV of the COF‐1/CB electrode (red line), measured in electrolyte with an additional 1 m MeOH (yellow line), and electrode after constant potential ORR for 24 h (blue line). Bulk COF‐1 particles synthesized by the conventional method (green line), commercial Pt/C (black line), control electrode consisting of CB and Nafion immersed in the monomer‐containing electrolyte, without electrolysis (purple line), and as cast (gray line) were used as a reference sample. e) Tafel plot of the COF‐1/carbon composite electrodes. f) RRDE measurement of the COF‐1/CB electrode.

The COF‐1/carbon samples were evaluated as organic electrocatalysts for facilitating ORR in a basic aqueous electrolyte. During the electrocatalytic performance tests, the COF‐1/MWCNT composite was prepared on a rotating disk electrode (RDE) with a GC surface. The cyclic voltammogram (CV) of the COF‐1/MWCNT electrode showed an enhanced reduction current corresponding to oxygen reduction when an O_2_‐saturated 0.1 mol dm^−3^ KOH aqueous solution was used as the electrolyte (**Figure** **5**a). Under an argon atmosphere, the COF‐1/MWCNT electrode displayed no significant redox peaks within the potential range of 0–1.5 V versus RHE, indicating that the COF‐1/MWCNT composite is electrochemically stable within this potential range. LSV corresponding to the ORR was conducted in the O_2_‐saturated solution at a rotation speed of 1600 rpm. The relationship between the thickness of the COF‐1 layer on MWCNT and ORR activity was investigated (Figure 5b). The COF‐1/MWCNT samples with COF‐1 thicknesses of 6.2, 13.1, and 22.2 nm exhibited onset potentials of 0.74, 0.66, and 0.63 V versus RHE, respectively. The control sample, consisting of MWCNT and Nafion, demonstrated a lower reduction current within the target potential range, highlighting the ability of COF‐1 to facilitate the electrochemical ORR. Although the COF‐1 compositions of these samples were not consistent, the trend between COF layer thickness and onset potential indicates that a thinner COF layer is more effective in enhancing ORR performance.

The onset potential of the COF‐1/MWCNT composite remained relatively low, likely due to the small specific surface area of the used MWCNT (15 m^2^ g^−1^). Consequently, the COF‐1/CB composite was tested as an ORR electrocatalyst. The LSV of the COF‐1/CB composite displayed an onset potential of 0.80 V versus RHE and reached a diffusion‐limited current in the high overpotential region (Figure 5c), following several optimizations of the loading amount of CB and electrolysis time (Figure S7, Supporting Information). From the Levich plot, the electron transfer number reached 3.56, suggesting that four‐electron reaction was the main pathway. The bulk COF‐1 sample, which formed sub‐micrometer‐scale aggregated particles, showed an onset potential of 0.64 V versus RHE (green line in Figure 5d). The control sample, prepared without electrolysis, showed slight improvement of onset potential (purple line in Figure 5d), indicating the electrolysis process is essential for the COF‐1 deposition. These results suggest that the surface‐selective COF synthesis method can enhance ORR properties. The hydrophobicity of the COF‐1/CB was investigated by contact angle measurement (Figure S8, Supporting Information). The water contact angle of COF‐1/CB composite film is 138 ± 4.9°, showing a hydrophobic surface. In a previous study, it was reported that the surface affinity to oxygen gas could enhance the ORR electrocatalyst property,^[^ ^39^, ^40^ ^]^ implying the deposition of the hydrophobic COF‐1 on support is an effective approach.

Additionally, the onset potential of the COF‐1/CB composite remained unchanged after continuous ORR for 24 h (blue line in Figure 5d), and the morphology of the COF‐1/CB composite was stable after 24 h of electrolysis (Figure S8, Supporting Information). The addition of methanol to the electrolyte did not alter the catalytic activity (yellow line in Figure 5d). In Tafel analysis (Figure 5e), the slope of COF‐1/CB, COF‐1/MWCNT, and bulk COF reference were 98, 110, 106 mV decade^−1^, respectively. The difference should correspond to the long‐range conductivity of the catalyst layer because the catalytic active site originates from the same COF‐1 structure. The selectivity between the four‐electron and two‐electron reduction pathways was investigated using a rotating ring‐disk electrode (RRDE) (Figure 5f). The electron transfer number was 3.64 for the COF‐1/CB electrode at 0.50 V versus RHE, indicating that the four‐electron reaction predominantly occurred during the ORR process. These findings demonstrate that the COF‐1/CB electrode serves as an effective electrocatalyst for ORR, characterized by low overpotential and high stability.

To further elucidate the reaction pathway, density functional theory (DFT)‐based simulations of the ORR on COF‐1 were conducted (Figure S10, Supporting Information). Previous studies have suggested that nitrogen atoms in π‐conjugated systems can act as active sites for electrocatalytic ORR.^[^ ^17^, ^18^, ^19^, ^20^ ^]^ COF‐1 contains two types of nitrogen atoms: those involved in imine bonds and those within the triazine ring. Calculations using the partial structure of COF‐1 were performed at the B3LYP+d3/6‐31G(d,p) level of theory, starting with two different initial positions for O_2_. The resulting free energy diagram indicated that the reaction pathway initiated by triazine‐N has a lower energy barrier than the pathway initiated by imine‐N. The suggestion from our DFT calculations coincides with the proposed mechanism reported by previous work.^[^ ^17^ ^]^

The onset potential of the COF‐1/CB composite is among the best reported for triazine‐N‐based organic ORR electrocatalysts (Figure S11 and Table S1, Supporting Information). Although some studies have reported superior properties, they often attribute the interaction with oxygen to other electron‐rich active sites, such as thiophene rings. This study demonstrates that nanometer‐scale controlled synthesis of COFs on nanocarbons can significantly improve electrocatalytic performance. The effectiveness of precise COF deposition techniques has been successfully demonstrated in this work.

## 3 Conclusion

An electrochemical nanoscale deposition method for imine‐type COFs has been developed. The key strategy for achieving nanometer‐scale control of the COF layer on nanocarbon supports is the precise control of the electrochemically generated acid based on programable electrolysis conditions. The COF/CB composite was evaluated as an ORR electrocatalyst, demonstrating superior performance among COF‐based electrodes containing triazine‐N active sites. The composite structure, featuring a single nanometer‐scale COF layer on nanocarbon, significantly enhances electrocatalytic ORR activity, even when the COF consists of typical heteroaromatic rings. This study demonstrates the effectiveness of the electrochemical COF deposition method and the design strategy for COF/carbon composite electrodes. Moreover, the proposed methodology for fabricating precise COF/carbon composites, especially containing optimized molecular structure to serve an effective active site, holds potential for improving electrode properties across a wide range of organic electrocatalyst applications.

## Conflict of Interest

The authors declare no conflict of interest.

## Supporting information



Supporting Information

## Data Availability

The data that support the findings of this study are available in the supplementary material of this article.
