# Supplementary material for: Electrochemically Controlled Deposition of Low‐Crystalline Covalent Organic Frameworks on Nanocarbon Electrode Toward Metal‐Free Oxygen Reduction Electrocatalyst
Source: Small. 2024 Dec 17;21(6):2410475. doi: 10.1002/smll.202410475 (PMC11817911; doi:10.1002/smll.202410475)
Supplement: Supplementary file 1 — Supporting Information [file SMLL-21-2410475-s001.docx]

**Supporting Information**

**Electrochemically Controlled Deposition of Low-Crystalline Covalent Organic Frameworks on Nanocarbon Electrode toward Metal-Free Oxygen Reduction Electrocatalyst**

Kosuke Sato*, Shinsuke Inagi*

Department of Chemical Science and Engineering, School of Materials and

Chemical Technology, Institute of Science Tokyo

4259 Nagatsuta, Midori-ku, Yokohama 226-8501, Japan

E-mail: sato.k.cx@m.titech.ac.jp; inagi@cap.mac.titech.ac.jp

**Contents**

| Experimental procedure | P. S2 |
| --- | --- |
| Characterizations of used carbon materials (Figure S1) | P. S4 |
| Synthesis and characterization of the reference COF-1 sample (Figure S2) | P. S5 |
| TGA of the COF-1/CB composite (Figure S3) | P. S6 |
| XRD profiles of the COF-1/carbon composite (Figure S4) | P. S7 |
| Thickness analysis on TEM images (Figure S5) | P. S8 |
| Thickness control on the COF-1/CB composite (Figure S6) | P. S9 |
| Optimization of the fabrication method for the COF-1/CB composite (Figure S7) | P. S10 |
| Contact angle study of COF-1/CB composite film (Figure S8) | P. S11 |
| COF/CB samples after durability test (Figure S9) | P. S12 |
| DFT calculation about ORR reaction pathway (Figure S10) | P. S13 |
| Comparison of the ORR properties with previous reports (Table S1 and Figure S11) | P. S14 |
| Supporting references | P. S16 |

**Experimental procedure**

**EGA-assisted synthesis of COF-1/carbon composite.** All reagents and materials were used as purchased without additional purification or treatment. Typically, 5 mg of carbon black (Cabot, Vulcan X-72, with 250 m g^−1^ of specific surface area) was dispersed in ethanol (0.95 cm^3^). Then, 0.05 cm^3^ of Nafion solution (Sigma-Aldrich, containing 5 wt.% resin) was added to the dispersion, which was sonicated for 10 min. The dispersion just after sonication was cast onto a glassy carbon (GC) substrate to fabricate the film, with the loading amount adjusted to 300 µg cm^−2^. A GC rotating disk electrode (φ = 5 mm) was used as the substrate for fabricating the COF-1/carbon composite. For thermogravimetric and XRD analyses, a GC plate (10 mm × 30 mm, masked to expose 10 mm × 10 mm of effective area) was used as the substrate. MWCNT (VGCF, Showa Denko, with 15 m^2^g^−1^ of specific surface area) was also used alternate of CB.

The carbon black-modified GC electrode was placed in a three-electrode electrochemical cell, equipped with a Pt wire as the counter electrode and an Ag/Ag^+^ electrode (0.01 mol dm^−3^ AgNO_3_ and 0.1 mol dm^−3^ TBAClO_4_ acetonitrile solution as the internal solution) as the reference electrode. A precursor DMF solution containing 10 mmol dm^−3^ of triaminophenyl-1,3,5-triazine (TCI, 98%), 15 mmol dm^−3^ of 2,5-dimethoxybenzene-1,4-dicarboxaldehyde (TCI, 98%), 2 mmol dm^−3^ of diphenyl hydrazine (Aldrich, 95%), and 100 mmol dm^−3^ of TBABF_4_ was used as the electrolyte. Electrolysis was conducted in current potential mode, set to 0.3 V vs. Ag/Ag^+^ for several seconds using a potentiostat (Biologic, SP-150). After electrolysis, the working electrode was kept in the electrolyte for 5 min to allow for ripening, then washed with DMF, acetone, and hexane. The COF/carbon composite on the electrode was dried at room temperature prior to characterization and measurement. Commercial Pt/C (10wt.% Pt, Sigma-Aldrich) and the carbon black-modified electrode without the electrochemical COF deposition, and the carbon black-modified electrode after immersed in precursor solution for 10 sec (without any electrolysis process) were used as control samples.

**Characterization.** The surface composition was analyzed by XPS using an ULVAC-PHI PHI VersaProbe 3 system. The morphologies of the samples were examined with a field-emission SEM, (Hitachi S-5500) and a TEM (JEOL JEM-1400). For TEM observation, the COF-1/carbon composite film was dispersed in ethanol using sonication, and a copper microgrid dipped into the dispersion was used for TEM sample preparation. The composition of the samples was determined by TGA using a Shimadzu DTG-60. High-order structures were analyzed by XRD with Cu-Kα radiation, utilizing a Rigaku Ultima-IV. Contact angle measurement was conducted by a contact-angle meter (Kyowa Interface Science, DMs-401).

**Electrochemical measurements.** A three-electrode setup in a beaker cell was employed for both electrochemical measurements and electrosynthesis.

The working electrode was a glassy carbon electrode (surface area: 0.07 cm^2^), with a platinum plate (surface area: 1 cm^2^) as the counter electrode, and an Ag/Ag^+^ electrode (0.01 mol dm^−3^ AgNO_3_ and 0.1 mol dm^−3^ TBAClO_4_ in acetonitrile as the internal solution) serving as the reference electrode. The electrolyte was 0.1 mol^−3^ TBABF_4_ in DMF. Target compounds were dissolved in the electrolyte to a concentration of 0.01 mol dm^−3^. Nitrogen bubbling was performed for 5 min prior to measurement. LSV was conducted at a sweep rate of 100 mV s^−1^, ranging from −0.5 V to 1.0 V vs. Ag/Ag^+^.

ORR measurement: A glassy carbon rod (3 mm diameter) and an Ag/AgCl electrode with a saturated KCl solution were used as the counter and reference electrodes, respectively. LSV was conducted using a potentiostat (BioLogic SP-150) at a scan rate of 10 mV s^−1^. The electrolyte was a 0.1 mol dm^−3^ KOH solution. The electrolyte was saturated with O_2_ by bubbling with O_2_ for 10 min before each experiment, and O_2_ flow was maintained during measurements. The conversion factor from Ag/AgCl to RHE is 0.958 V in a 0.1 mol dm^−3^ KOH solution, as determined by Equation (1). The pH value of the electrolyte was measured using a pH meter (HORIBA F-71S).


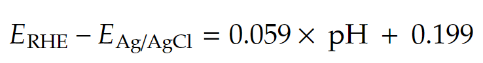
… eq-(1)

A rotating ring disk electrode (RRDE, PINE Research AFE6R2GCPT) was used to estimate the electron transfer number. During LSV measurements, the RRDE was rotated at 400 rpm, and the ring electrode (Pt) was maintained at −0.1 V vs. Ag/AgCl. The electron transfer number (*N*_ET_) was calculated using Equation (2), with a collection efficiency (*E*_collect_) of 38.3% for the RRDE used.


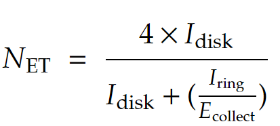
… eq-(2)

**Statistical Analysis.** The layer thickness and particle diameter data were analyzed manually from SEM and/or TEM images. The contact angle was obtained from the waterdrop images at different sites of each sample. Entire datapoints (*N*) were 20 for TEM images, 30 for SEM images, 10 for contact angle. These data obtained through statical process were represented as (average value) ± (standard deviation).

**Characterizations of used carbon materials**

**
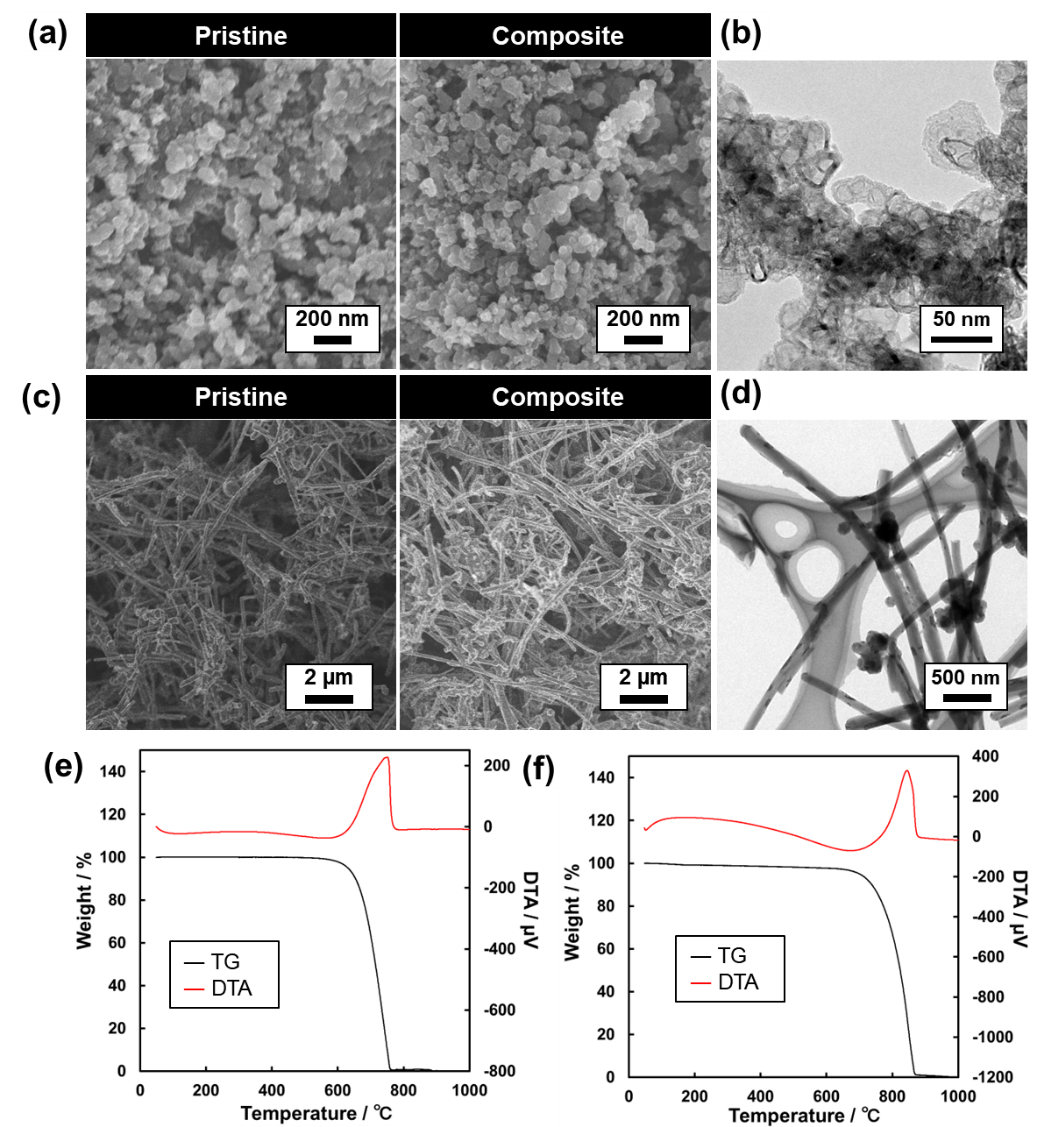
**

**programmable** (a) Comparison of SEM images of CB and COF-1/CB film. (b) TEM image of CB. (c) Comparison of SEM images of MWCNT and MWCNT/CB film. (d) TEM image of MWCNT. (e,f) TG curve and DTA profile of CB (e) and MWCNT (f). The measurement conducted in air with 20 °C min^−1^ of heating rate.

Based on SEM images, both the COF-1/CB and COF-1/MWCNT composite maintained the original shape after electrolysis, indicating that the surface-selective COF deposition occurred without generation of bulk COF particles. In TG curve, CB and MWCNT exhibited 639 °C and 701 °C as 5% weight-loss temperature, respectively.

**Synthesis and characterization of the reference COF-1 sample**


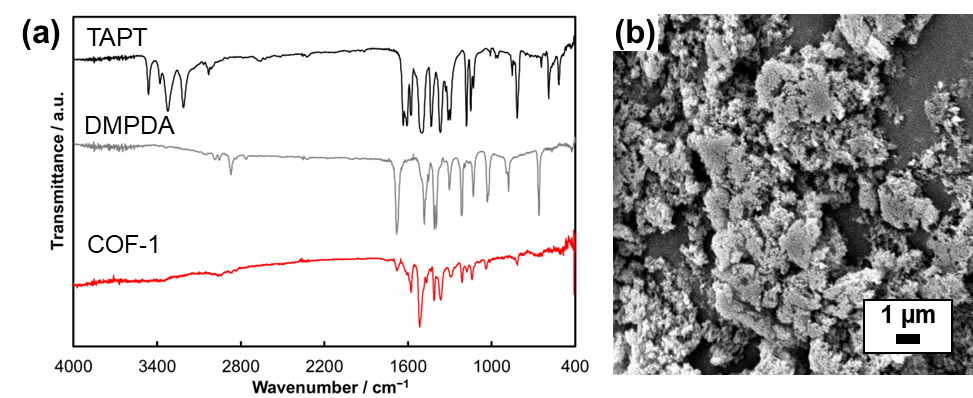


**Figure S2**. (a) FT-IR spectra of TAPT, DMPDA, and COF-1. (b)SEM image of the reference COF-1 sample.

The COF-1 sample was fabricated via typical liquid-phase system^s1^ for a comparison. Based on the FT-IR spectra, the generation of COF-1 was confirmed. The reference COF-1 sample showed agglomerated particles with sub-µm diameter.

**TGA of the COF-1/CB composite**


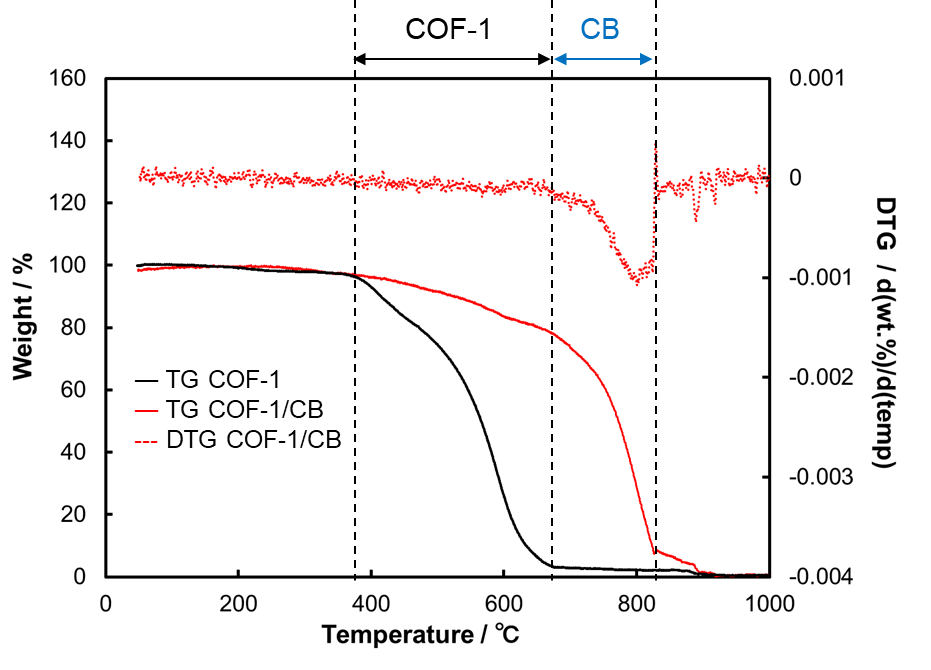


**Figure S3.**  TG curve of COF-1 and COF-1/CB, and DTG profile from the TG curve of COF-1/CB. Measurements were done in air flow with 20 °C min^−1^ of temperature rising rate.

The COF-1 sample showed the weight degradation in range of 370–670 °C. The COF-1/CB composite showed the weight degradations in range of 370–670 °C and in range of 670–830 °C. Ther latter should be derived from a combustion of CB (Figure S1e). The DTG profile of the COF-1/CB showed a single peak in the corresponding temperature range. Based on the TG curves, the COF-1/CB sample was composed of 21 wt.% COF-1.

According to TEM image, the CB/COF-1 has the core-shell structure: 60 nm CB core and 6 nm COF-1 shell. The calculated composition based on this model structure is 20 wt.%, on the assumption of their density values, d = 1.7 for CB and d = 1.3 for COF-1.

**XRD profile of the COF-1/carbon composite**


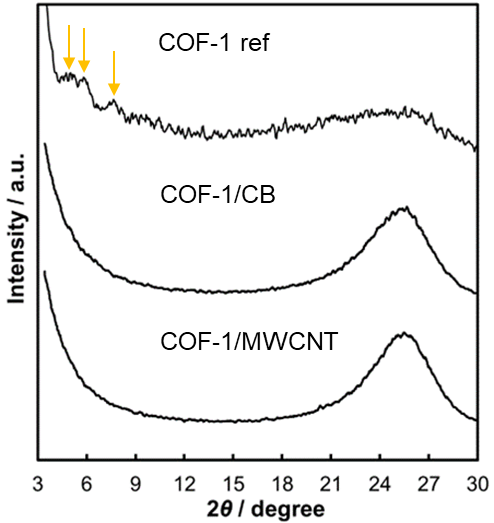


**Figure S4.**  TG curve of the reference COF-1, COF-1/CB, and COF-1/MWCNT. The composite samples were fabricated on the GC substrate (10 mm×10 mm) with 0.3 V vs. Ag/Ag^+^ of applied potential for 30 sec. The broad peak around 2*θ* = 25° was derived from the GC substrate.

The reference COF-1 sample showed diffraction peaks at 5.2, 6.3, and 8.0°, derived from (200), (210), (220) plane of its crystalline ordered structure. However, the COF-1/CB and COF-1/MWCNT did not show the corresponding peaks.

**Thickness analysis on TEM images**


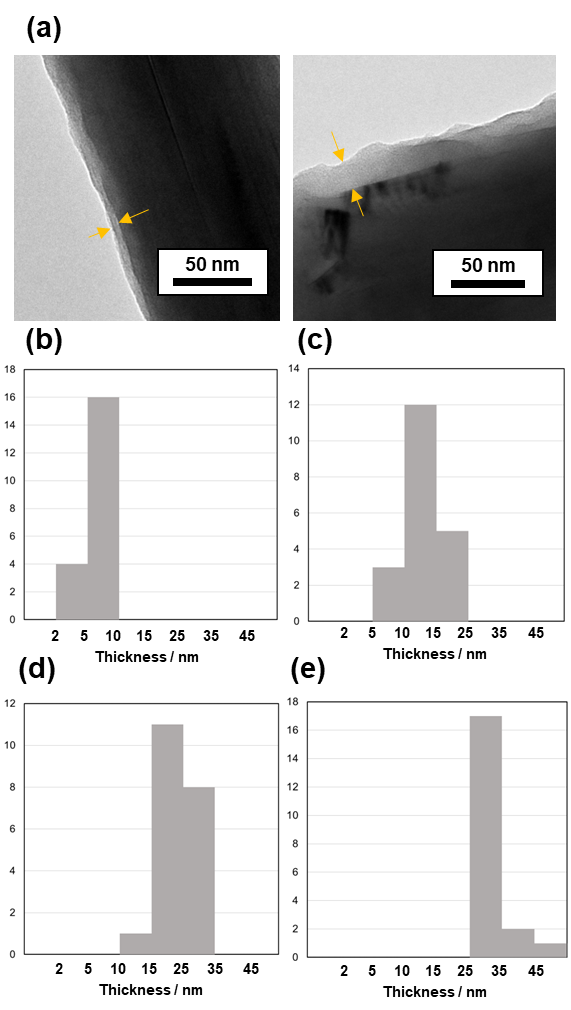


**Figure S5.** Detailed TEM image analysis for COF-1/MWCNT. (a) example TEM image of COF-1/MWCNT composite, the COF-1 layer was pointed by yellow arrow. (b–e) Histograms describing entire dataset of COF-1 layer thickness. (b) 10 sec, (c) 30 sec, (d) 60 sec, (e) 90 sec, N=20.

**Thickness control of the COF-1/CB composite**


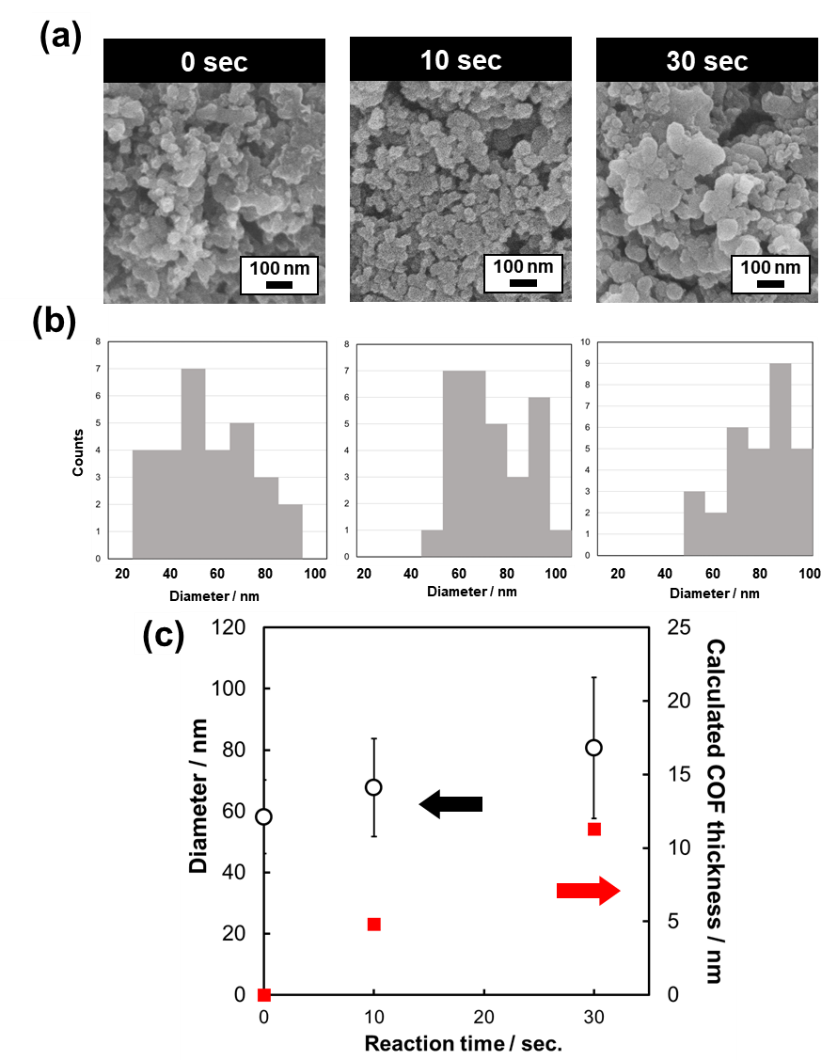


**Figure S6.** (a) SEM images of the COF-1/CB composite with various electrolysis time, (b) Relationship between the electrolysis time and the observed diameter of the COF-1/CB composite, N=30.

When CB was used as support, COF-1 was generated on the surface of CB. The diameter of the composite increased with the increase of the reaction time.

**Optimization of fabrication condition for the COF-1/CB composite**


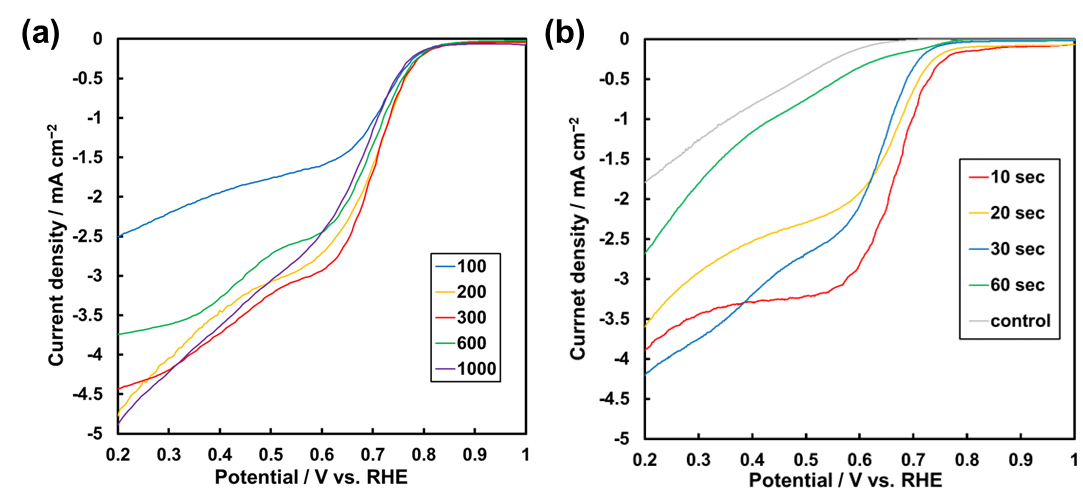


**Figure S7.** LSVs of COF-1/CB composite electrode measured in O_2_-saturated 0.1 mol dm^−3^ KOH aqueous solution at a rotating speed of 1600 rpm. (a) optimization of loading amount. 100, 200, 300, 600, 1000 µg cm^−2^ were tested. (b) optimization of the electrolysis time at COF-1 deposition step.

When CB was loaded at 100 µg cm⁻², the cast film did not form uniformly, and COF-1 was generated partially. The bare GC area caused the low current density. The electrodes with loading amounts of 200–600 µg cm⁻² showed similar activity, with the best condition determined to be 300 µg cm⁻². When CB was loaded at 1000 µg cm⁻², the onset potential was slightly lowered, probably because the large thickness of the composite layer prevented efficient electron transport. The electrolysis time was adjusted to 10 sec, to produce thinner COF-1 layer.

**Contact angle study of COF-1/CB composite film**

**
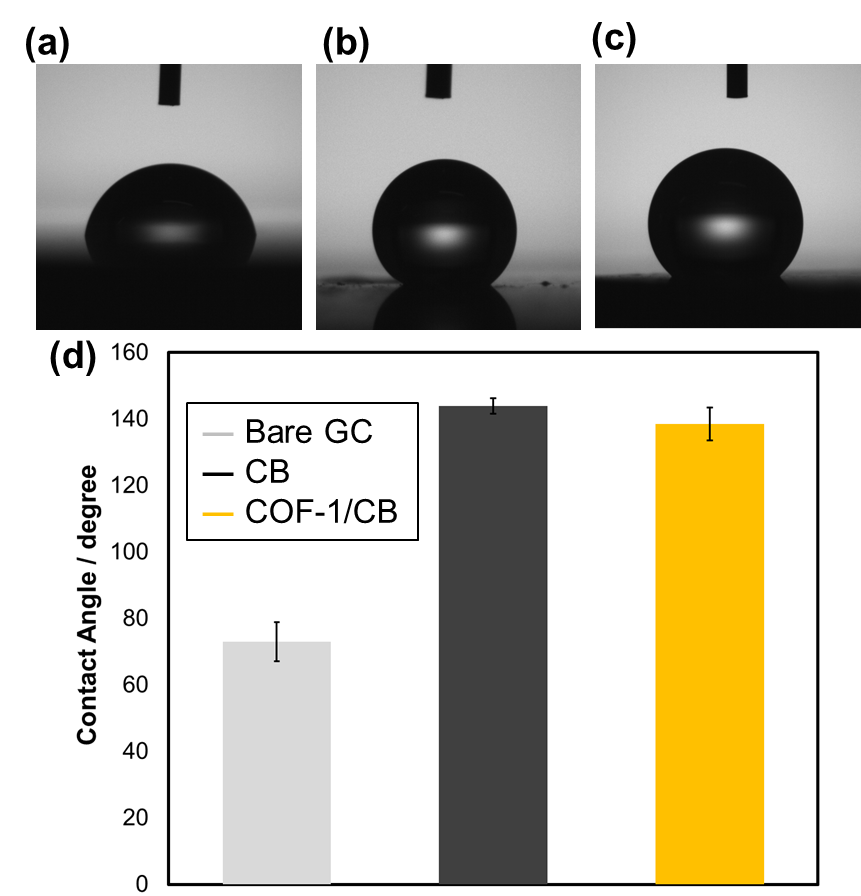
**

**Figure S8.** Contact angle measurement of COF-1/CB composite. (a–c) Images of a water droplet on substrate: GC plate (a), casted film of CB (b), and COF-1/CB composite film (c). (d) Summery of the obtained contact angle data, N=10. The CB and COF-1/CB samples were fabricated by the same method except using GC plate as substrate instead of the RDE.

The CB film showed a hydrophobic surface with contact angle of 143±2.3°. After the electrochemical COF-1 deposition, the contact angle was slightly decreased to 138±4.9°.

**the COF-1/CB sample after durability test**


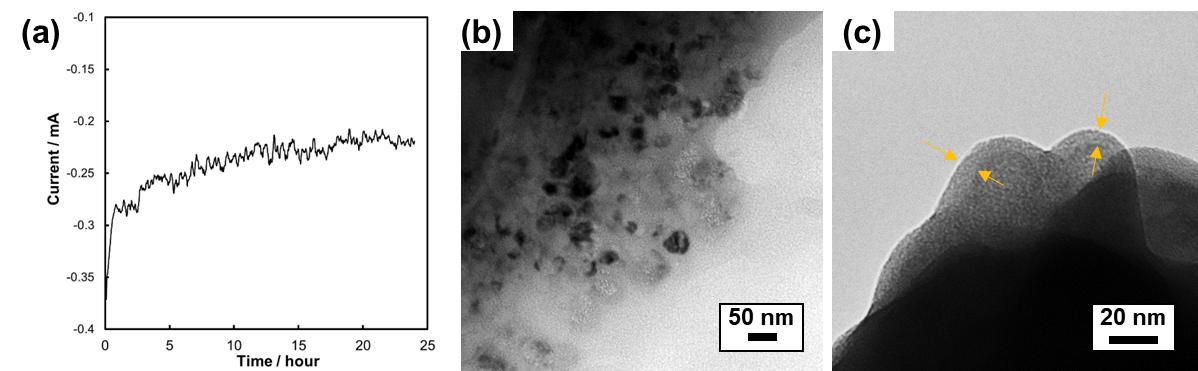


**Figure S9.** (a) Chronoamperogram of the continuous ORR on the COF-1/CB electrode. The electrode set at 0.6 V vs. RHE for 24 h during air flow. The observed noise and current decrease were caused by the air bubble. (b) TEM images of the COF-1/CB composite after continuous ORR at 0.6 V vs. RHE for 24 h. The COF-1 layer was emphasized by yellow arrows. The core-shell structure of COF-1/CB composite was maintained.

**DFT calculations about ORR reaction pathway**


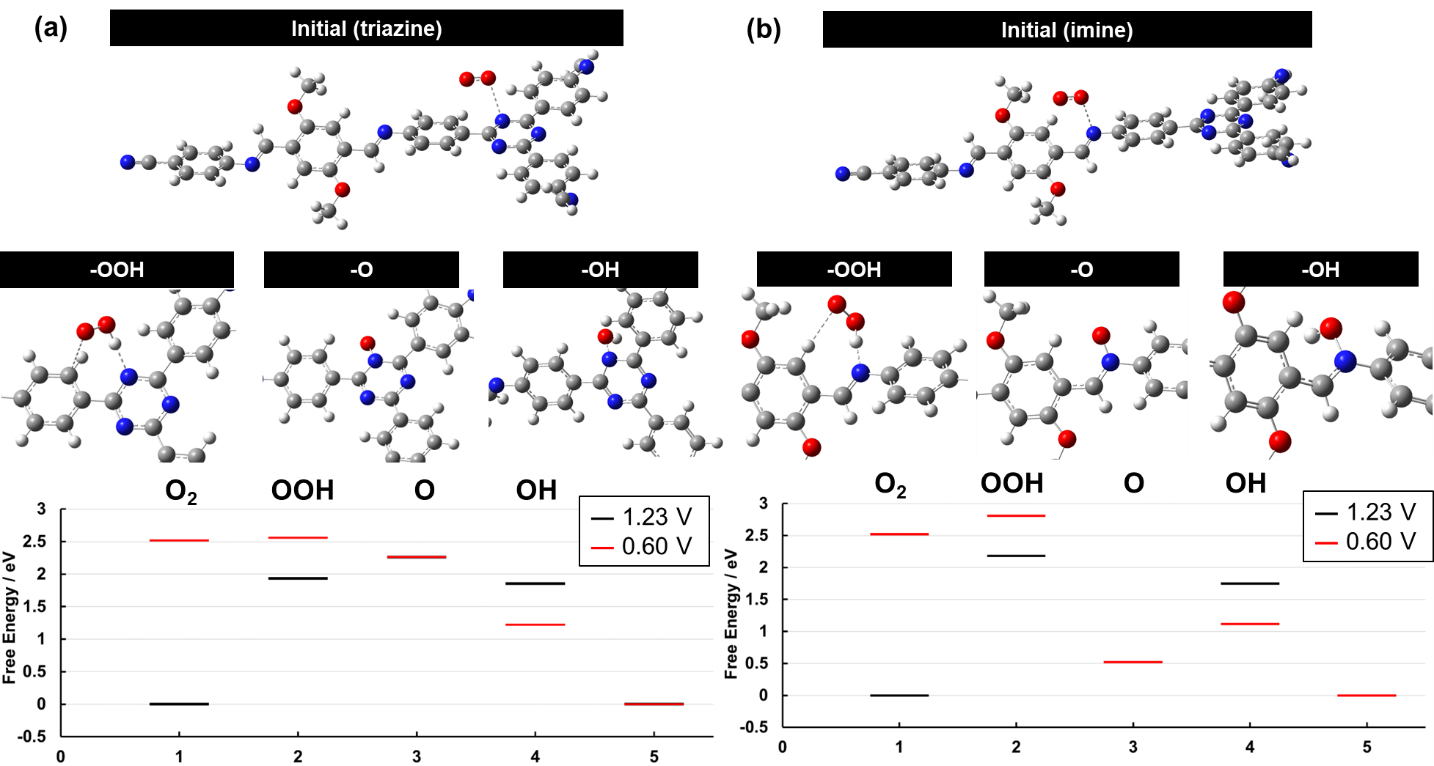


**Figure S10.** DFT calculation-based simulation of ORR through (a) triazine-N initiated and (b) imine-N initiated reaction path. The calculation was conducted on B3LYP+d3/6-31G(d,p) level of theory by using Gaussian 16.^s2^

The free energy diagram was calculated from the Gibbs energy of the intermediates, reported value from literature, and electron potential according to following eq (1−4).^s3,s4^

${\Delta G}_{O_{2}\_OOH}=G_{OOH*}+G_{H_{2}O}-G_{\mathrm{cat}}-{3G}_{\mathrm{OH}^{-}}-3eU$ … **eq-(1)**

${\Delta G}_{OOH\_O}=G_{O*}-G_{OOH*}+G_{\mathrm{OH}^{-}}-eU$ … **eq-(2)**

${\Delta G}_{O\_OH}=G_{OH*}-G_{O*}-G_{H_{2}O}+G_{\mathrm{OH}^{-}}-eU$ … **eq-(3)**

${\Delta G}_{OH\_\mathrm{OH}^{-}}=-G_{OH*}+G_{\mathrm{cat}}+G_{\mathrm{OH}^{-}}+eU$ … **eq-(4)**

Based on the free-energy diagram, the triazine-N initiated pathway can reach down-hill mode around 0.6 V vs. RHE. The imine-N initiated pathway still has Gibbs energy barrier on the first step at the condition. In the triazine-N initiated pathway, the -OOH state stabilized by both N atom and the aromatic C atom at γ-position to the N atom, although only N atom contributed the interaction in the case of the imine-N pathway.

**Comparison of the ORR properties with previous works reporting triazine-type COF**

**Table S1** Relationships of onset potential and electron transfer number on ORR among previously reported organic electrode containing triazine ring.

| **ID** | **Plot color** | **Active site** | **Onset potential**  **/ V vs. RHE** | **N_ET_**  **/ -** | **Ref.** |
| --- | --- | --- | --- | --- | --- |
| **1** | **●** | **Triazine** | **0.78** | 3.63 | r1 |
| **2** | **●** | **Triazine**  **+imine** | **0.77** | 3.52 | r2 |
| **3** | **●** | **Triazine** | **0.81** | 3.67 | r3 |
| **4** | **●** | **Triazine** | **0.75** | 3.67 | r3 |
| **5** | **●** | **Triazine** | **0.78** | 3.42 | r3 |
| **6** | **●** | **Triazine** | **0.77** | 3.43 | r3 |
| **7** | **●** | **Triazine** | **0.70** | 2.64 | r4 |
| **8** | **●** | **Triazine** | **0.75** | 2.6 | r4 |
| **9** | **●** | **Triazine** | **0.71** | 2.95 | r5 |
| **10** | **●** | **Triazine** | **0.77** | 3.5 | r5 |
| **11** | **●** | **Triazine** | **0.77** | 3.62 | r5 |
| **12** | **●** | **Triazine** | **0.80** | 3.57 | r5 |
| **13** | **▲** | **Imine** | **0.74** | 3.30 | r6 |
| **14** | **▲** | **Azo bond** | **0.81** | 3.38 | r6 |
| **15** | **▲** | **Heptazine**  **+ Triazine** | **0.83** | 3.90 | r7 |
| **16** | **□** | **Thiophene** | **0.80** | 3.81 | r8 |
| **17** | **□** | **Thiophene** | **0.75** | 3.46 | r8 |
| **18** | **□** | **Imide** | **0.75** | 3.50 | r9 |
| **19** | **□** | **Imide** | **0.78** | 3.60 | r10 |
| **20** | **□** | **Triphenylmethane** | **0.79** | 3.62 | r11 |
| **21** | **□** | **Fluorene** | **0.82** | 3.88 | r12 |
| **22** | **□** | **Selenadiazole** | **0.78** | 3.65 | r13 |
| **This work** | **●** | **Triazine** | **0.80** | **3.64** |  |

The electrocatalytic ORR properties of COF-1/CB are comparable to that of the previous studies reporting triazine-based organic electrocatalyst.


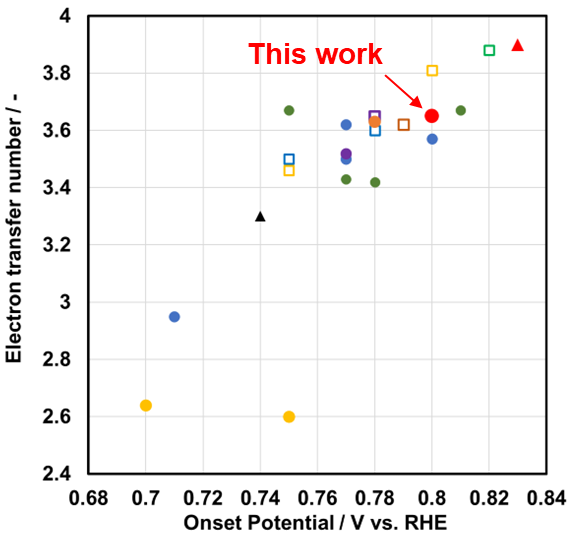


**Figure S11** Relationships of onset potential and electron transfer number on ORR electrocatalytic performance among previously reported structure-defined organic electrode.

**Supporting references**

[s1] R. Gomes, P. Bhanja, A. Bhaumik, *Chem. Commun.* **2015**, *51*, 10050–10053.

[s2] Gaussian 16, Revision C.01

M. J. Frisch, G. W. Trucks, H. B. Schlegel, G. E. Scuseria, M. A. Robb, J. R. Cheeseman, G. Scalmani, V. Barone, G. A. Petersson, H. Nakatsuji, X. Li, M. Caricato, A. V. Marenich, J. Bloino, B. G. Janesko, R. Gomperts, B. Mennucci, H. P. Hratchian, J. V. Ortiz, A. F. Izmaylov, J. L. Sonnenberg, D. Williams-Young, F. Ding, F. Lipparini, F. Egidi, J. Goings, B. Peng, A. Petrone, T. Henderson, D. Ranasinghe, V. G. Zakrzewski, J. Gao, N. Rega, G. Zheng, W. Liang, M. Hada, M. Ehara, K. Toyota, R. Fukuda, J. Hasegawa, M. Ishida, T. Nakajima, Y. Honda, O. Kitao, H. Nakai, T. Vreven, K. Throssell, J. A. Montgomery, Jr., J. E. Peralta, F. Ogliaro, M. J. Bearpark, J. J. Heyd, E. N. Brothers, K. N. Kudin, V. N. Staroverov, T. A. Keith, R. Kobayashi, J. Normand, K. Raghavachari, A. P. Rendell, J. C. Burant, S. S. Iyengar, J. Tomasi, M. Cossi, J. M. Millam, M. Klene, C. Adamo, R. Cammi, J. W. Ochterski, R. L. Martin, K. Morokuma, O. Farkas, J. B. Foresman, D. J. Fox, Gaussian, Inc., Wallingford CT, 2019.

[s3] Y. Jiao, Y. Zheng, M. Jaroniec, S. Z. Qiao, *J. Am. Chem. Soc.* **2014**, *136*, 4394–4403.

[s4] J. K. Nørskov, J. Rossmeisl, A. Logadottir, L. Lindqvist, J. R. Kitchin, T. Bligaard, H. Jónsson, *J. Phys. Chem. B* **2004**, *108*, 17886–17892.

――Papers reporting organic ORR electrode composed of triazine rings and/or other aromatic ring

[r1] J. Liu, Y. Hu, J. Cao, *Catal. Commun.* **2015**, *66*, 91–94.

[r2] T. Boruah, S. K. Das, G. Kumar, S. Mondal, R. S. Dey, *Chem. Commun.* **2022**, *58*, 5506–5509.

[r3] 1J. Chang, C. Li, X. Wang, D. Li, J. Zhang, X. Yu, H. Li, X. Yao, V. Valtchev, S. Qiu, Q. Fang, *Nano-Micro Lett.* **2023**, *15*, 159.

[r4] W. Yu, S. Gu, Y. Fu, S. Xiong, C. Pan, Y. Liu, G. Yu, *J. Catal*. **2018**, *362*, 1–9.

[r5] T. Sönmez, J. Uecker, H. H. Hamzah, R. Palkovits, *Int. J. Hydrogen Energy* **2024**, *71*, 588–599.

[r6] X. Li, S. Yang, M. Liu, X. Yang, Q. Xu, G. Zeng, Z. Jiang, *Angew. Chem. Int. Ed.* **2023**, *62*, e202304356.

[r7] J. Liu, C. Wang, Y. Song, S. Zhang, Z. Zhang, L. He, M. Du, *J. Colloid Interf. Sci.* **2021**, *591*, 253–263.

[r8] D. Li, C. Li, L. Zhang, H. Li, L. Zhu, D. Yang, Q. Fang, S. Qiu, X. Yao, *J. Am. Chem. Soc.* **2020**, *142*, 8104–8108.

[r9] M. Martínez-Fernández, E. Martínez-Periñán, S. Royuela, J. I. Martínez, F. Zamora, E. Lorenzo, J. L. Segura, *Appl. Mater. Today* **2022**, *26*, 101384.

[r10] S. Royuela, E. Martínez-Periñán, M. P. Arrieta, J. I. Martínez, M. M. Ramos, F. Zamora, E. Lorenzo, J. L. Segura, *Chem. Commun.* **2019**, *56*, 1267–1270.

[r11] S. Wu, M. Li, H. Phan, D. Wang, T. S. Herng, J. Ding, Z. Lu, J. Wu, *Angew. Chem. Int. Ed.* **2018**, *57*, 8007–8011.

[r12] Q. Cao, L. Wan, Z. Xu, W. Kuang, H. Liu, X. Zhang, W. Zhang, Y. Lu, Y. Yao, B. Wang, K. Liu, *Adv. Mater.* **2023**, *35*, e2210550.

[r13] J. Jia, J. Li, S. Ma, Z. Zhang, X. Liu, *Macromol. Rapid Commun.* **2023**, *44*, e2200717.
